# A Novel Hybrid Model Based on a Feedforward Neural Network and One Step Secant Algorithm for Prediction of Load-Bearing Capacity of Rectangular Concrete-Filled Steel Tube Columns

**DOI:** 10.3390/molecules25153486

**Published:** 2020-07-31

**Authors:** Quang Hung Nguyen, Hai-Bang Ly, Van Quan Tran, Thuy-Anh Nguyen, Viet-Hung Phan, Tien-Thinh Le, Binh Thai Pham

**Affiliations:** 1 Thuyloi University, Hanoi 100000, Vietnam; 2 University of Transport Technology, Hanoi 100000, Vietnam; banglh@utt.edu.vn (H.-B.L.); quantv@utt.edu.vn (V.Q.T.); binhpt@utt.edu.vn (B.T.P.); 3 University of Transport and Communications, Ha Noi 100000, Vietnam; phanviethung@utc.edu.vn; 4 Institute of Research and Development, Duy Tan University, Da Nang 550000, Vietnam

**Keywords:** concrete-filled steel tube column, machine learning, neural network, one step secant algorithm, optimization

## Abstract

In this study, a novel hybrid surrogate machine learning model based on a feedforward neural network (FNN) and one step secant algorithm (OSS) was developed to predict the load-bearing capacity of concrete-filled steel tube columns (CFST), whereas the OSS was used to optimize the weights and bias of the FNN for developing a hybrid model (FNN-OSS). For achieving this goal, an experimental database containing 422 instances was firstly gathered from the literature and used to develop the FNN-OSS algorithm. The input variables in the database contained the geometrical characteristics of CFST columns, and the mechanical properties of two CFST constituent materials, i.e., steel and concrete. Thereafter, the selection of the appropriate parameters of FNN-OSS was performed and evaluated by common statistical measurements, for instance, the coefficient of determination (*R*^2^), root mean square error (*RMSE*), and mean absolute error (*MAE*). In the next step, the prediction capability of the best FNN-OSS structure was evaluated in both global and local analyses, showing an excellent agreement between actual and predicted values of the load-bearing capacity. Finally, an in-depth investigation of the performance and limitations of FNN-OSS was conducted from a structural engineering point of view. The results confirmed the effectiveness of the FNN-OSS as a robust algorithm for the prediction of the CFST load-bearing capacity.

## 1. Introduction

Concrete-steel composite structure has been the subject of extensive researches and widely applied in the construction industry as a result of the efficiency in combining the two most commonly used materials: concrete and steel [1]. Concrete filled steel tube (CFST) column is a type of composite structure that can replace traditional column structures, such as reinforced concrete columns or steel columns [2]. The CFST column could take full advantage of the bearing capacity of concrete and steel by overcoming the weaknesses of each component while working simultaneously in the structure. Moreover, the CFST columns exhibit many advantages, especially the profit of the ductility, associated with the steel structures, and the stiffness of the concrete system. Thereby, the construction costs could be reduced to the lowest level [3].

The typical characteristic of CFST is that the concrete material stuffing in the steel pipe hinders local instability of the pipe wall while subjected to compression. Besides, the steel in the CFST section is much more significant than that of reinforced concrete, positioned at the farthest end of the section. This could significantly increase the bearing capacity of the structure [4,5,6,7,8]. Previous studies [9,10,11,12,13] also showed that the CFST column has high flexibility, high energy absorption, and high reliability when used for earthquake-resistant buildings. Moreover, it is able to reduce the impact on the environment by eliminating formwork, and the steel pipes could be reused, or using high-strength concrete with recycled materials. Compared with standard steel columns, CFST columns are especially useful when subjected to compression. Therefore, in a load-bearing structure system, it is recommended to use CFST columns for compressive structures [13,14,15]. With such bearing characteristics, the cross-section of the CFST column is usually in the form of circle, square, rectangle, or ellipse [13]. For load-bearing components in two uneven directions, the cross-section of CFST is usually chosen in the form of rectangular or elliptical shapes [8,12,16,17].

So far, the CFST has been widely used around the world in various types of structures such as compressive columns in tall buildings, steel pipe arch bridges, piles, transmission towers, and bracing members in buckling restrained frames [2,12,18]. Therefore, the CFST column calculation regulations have been included in several design standards, such as the “Load and resistance factor design LRFD specification for structural steel buildings”, issued by the American Steel Works Institute, ANSI/AISC. 360-10 [19], Canadian Standards: Limit state design of steel structures, CAN/CSAS16.1-M94 [20], Eurocode 4 (EC4) [21], Australian standard AS 5100 [22], Chinese standard CECS 28-2012 [23], and Japanese standard JIS G 3192: 2005 [24]. In addition, an important number of empirical studies and numerical works have been carried out, based on the mechanical properties of the CFST column with different cross-sectional forms under the influence of axial load. Studies by Liu et al. [12,18], Chitawadagi et al. [25], Schneider [26], Uy [6], Sakino et al. [27], and many other studies [10,28,29,30,31] related to rectangular CFST columns with axial load have shown that the bearing capacity of CFST columns depends on many factors, such as the changes in the pipe wall, the thickness of steel pipe, concrete strength, cross-sectional area of steel pipe, steel pipe length, effects of concrete compaction, effective load conditions and boundary conditions. However, in the above studies, there are still some limitations, such as the difference between standard and experimental results [32], simplified methods in design codes are not suitable for materials of high strength [33], the process of testing axial compressions is time-consuming and labor-intensive. It is also difficult in numerical methods to consider all the complex conditions and properties of the materials used [1]. At the same time, these methods have not yet generally considered the factors affecting the load capacity of CFST column. Therefore, it is necessary to develop a consistent and effective method to design CFST columns.

In recent decades, artificial intelligence (AI) or machine learning has progressively become prevalent and applied in miscellaneous engineering fields [34,35,36,37,38,39,40,41,42,43,44]. The artificial neural network (ANN), a well-known AI algorithm, has been widely used and applied to construction engineering. Various contributions have demonstrated the potential of ANN in predicting the behavior of structural members and materials in the field of mechanical engineering, especially in the field of construction [45,46,47,48,49,50]. Regarding the CFST columns, many studies related to AI have been conducted to study the CFST behavior under different conditions. In the study of Al-Khaleefi et al. [51] and Wang et al. [52], the relationship of fire resistance and load-deformation of the CFST columns with different dimensions and parameters was predicted by ANN model. The load-bearing capacity of CFST under the effect of the axial load has also been predicted based on ANN models in the studies of Du et al. [33] or Sarir et al. [53]. However, the prediction capability of the proposed ANN model still needs further improvements. Moreover, the performance and limitations of ANN have not been studied, especially from a structural engineering point of view.

Therefore, this study focused on the development of an AI model based on a feedforward neural network (FNN) and one-step secant (OSS) algorithm to predict the load-carrying capacity of the rectangular CFST columns under axial loading. The OSS algorithm was used in the training phase of the FNN model to optimize the weights and biases associated with the neurons in the hidden layer for developing a hybrid model (FNN-OSS), aiming at a better prediction of the load-bearing capacity of rectangular CFST members. To this aim, a database consisted of 422 instances was collected from published works in the literature. The input variables in the database contained the geometrical characteristics of CFST columns, and the mechanical properties of two CFST constituent materials, i.e., steel and concrete. The parameters of FNN and OSS were first carefully selected, following by the evaluation of the performance of the FNN-OSS model. Next, the prediction capability of the best FNN-OSS structure was evaluated in local and global analyses. Finally, discussions and limitations on the robustness of the proposed FNN-OSS model were given through the prediction in function of different classes of input variables.

## 2. Material and Methods

### 2.1. Database Construction

**Figure 1:**
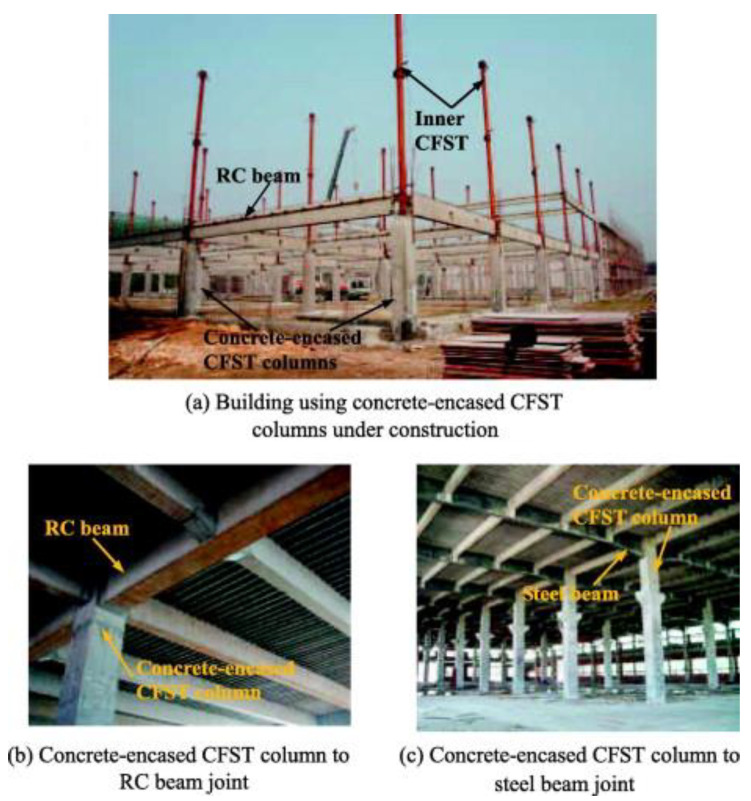
Applications of rectangular CFST columns in practical construction (reproduced with permission from Liao et al. [54]).

Composite CFST columns have been widely employed in various practical constructions as shown in Figure 1. The role of these columns is important as they support all the weight of the entire structure above. If the stability of even one structural component is not guaranteed, then the risk of damage to the structure is significant.

**Figure 2:**
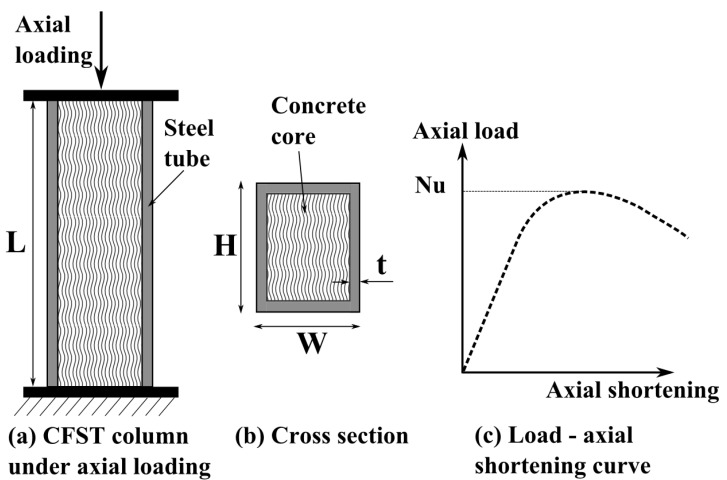
Diagram of rectangular CFST column: (**a**) under axial loading, (**b**) cross-section, and (**c**) load-axial shortening curve for determination of load-carrying capacity.

Various laboratory experiments have been performed in the literature to measure the load-bearing capacity of rectangular CFST columns. As set forth in the literature, the experimental process followed the steps below [55,56]: (i) design; (ii) processing of steel tube (welded or cold formed steel plates); (iii) production of concrete; (iv) manufacture of composite members; and (v) loading and measurement (see Figure 2 for schematic description of the test).

**Figure 3:**
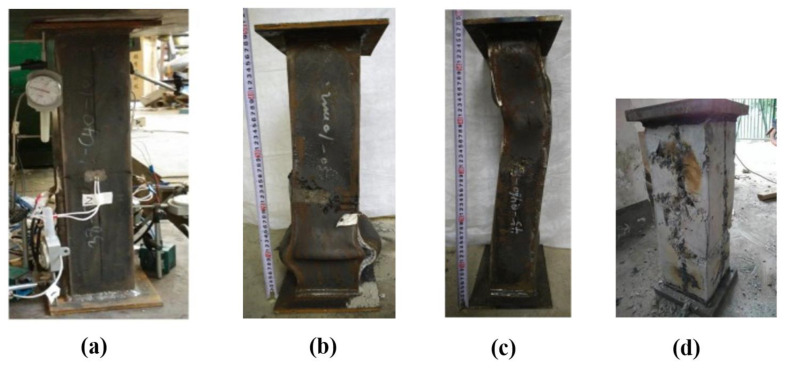
Experimental tests on CFST columns: (**a**) typical test setup and instrumentations (linear varying displacement transducers and strain gages were used to record the variations of displacement and strains) (represented with permission from Du et al. [55]), (**b**) local outward buckling of steel tube (represented with permission from Du et al. [55]), (**c**) overall buckling failure of slender column (represented with permission from Du et al. [55]), and (**d**) damage of concrete core (represented with permission from Lyu et al. [61]).

In this study, 422 tests on axially loaded rectangular CFST columns were gathered from the available literature. The selection of tests was based on the following criteria (see Figure 3a for typical test setup and instrumentations):

- Only monotonic uniaxial test was collected;
- The samples were fully loaded (both steel and concrete);
- Steel reinforcement, shear stub and tab stiffeners were not included in the samples.

In addition, a hypothesis was made such that the influence of initial geometric imperfections and residual stress was negligible compared to the major geometric parameters and mechanical properties of the constituent materials [57]. Diagram of CFST column under compressive loading is presented in Figure 2. Figure 2a,b show geometrical parameters of the column such as cross-sectional height and width, thickness of steel tube, and length of column. The strength of constituent materials is characterized through yield strength for steel and cylindrical compressive strength for concrete. The load-bearing capacity *N_u_* of the column is determined as shown in Figure 2c.

Typical damages of CFST columns are presented in Figure 3b for local outward buckling, Figure 3c for overall buckling failure, and Figure 3d for concrete core. In the presence of a concrete core, local outward buckling failure of the external steel was observed in all specimens, as shown in Figure 3b. This is the same as that observed by other investigations such as Han and Yao [58], Yan et al. [59]. On the other hand, the concrete core underwent shear failure (see Figure 3d). Slender CFST columns may fail through overall flexural buckling, together with (minor) local outward bulges (see Figure 3c). In several tests, tensile fractures were also observed in the steel wall [55,60] (see Figure 3b), because the tube was formed by welding.

**Figure 4:**
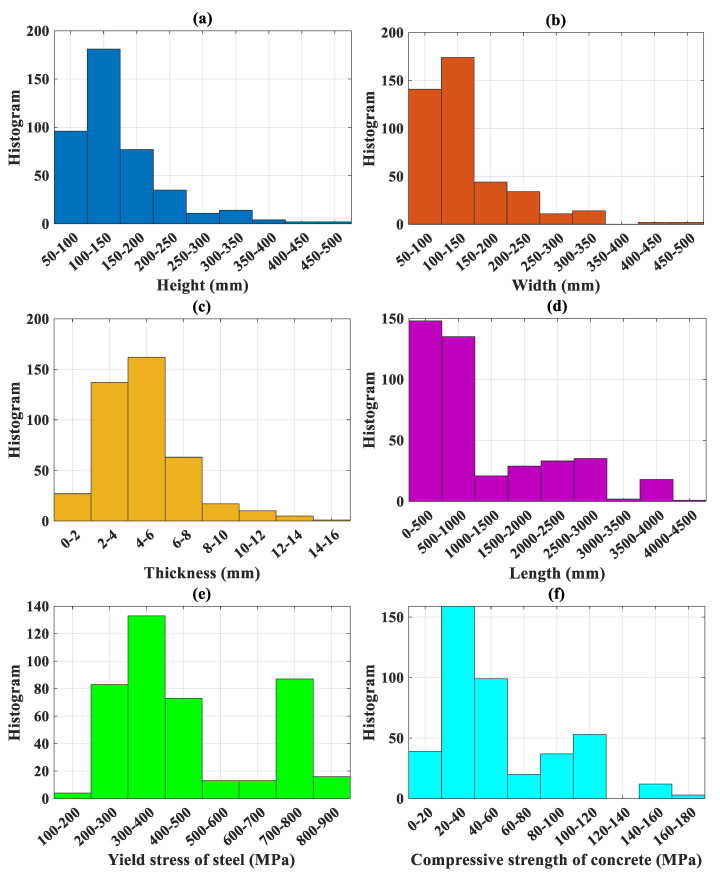
Classification of variables used in this study including the corresponding number of data: (**a**) height, (**b**) width, (**c**) thickness, (**d**) length, (**e**) steel yield stress and (**f**) concrete compressive strength.

**Figure 5:**
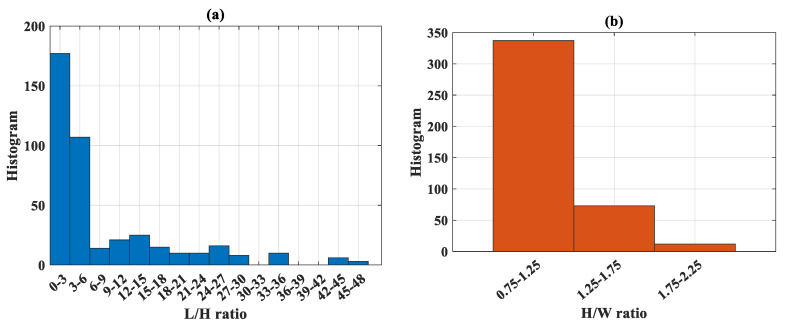
Classification of (**a**) *L*/*H* ratio and (**b**) *H*/*W* ratio, including the corresponding number of data.

The details of 422 experimental results on CFST structures are summarized in Table 1. Table 2 shows the initial statistical analysis regarding the database, including notation, unit, min, quantile, max, average, standard deviation, and coefficient of variation of all variables in the database. The input variables considered were the height of cross-section (denoted as *H*), the width of cross-section (denoted as *W*), the thickness of steel tube (denoted as *t*), the length of CFST column (denoted as *L*), the yield stress of steel (denoted as *f_y_*) and the compressive strength of concrete (denoted as *f’_c_*). The load-carrying capacity (denoted as *N_u_*) was considered as the output of the problem. Figure 4 displays the classification of variables used in this study, including the number of data and the distribution of values. Figure 5 displays the classification of the type of structures in highlighting the *L*/*H* and *H*/*W* ratios. Table 3 shows the details of classification regarding the *L*/*H* ratio, *H*/*W* ratio with the type of steel tube.

The 422 data used in this work were randomly divided into two sub-datasets (under a uniform distribution), 295 first configurations (70%) were served for training the model and 127 last configurations (30%) were served as the testing part. This 70–30 ratio was selected as recommended by Sharma et al. [94] and Salcedo-Sanz et al. [95] in order to ensure the effectiveness in the learning and testing processes.

### 2.2. Methods Used

#### 2.2.1. Feedforward Neural Network (FNN)

An artificial neural network (ANN) is a model/algorithm for information processing based on biological neuron systems. It is built on the basis of many elements (called neurons), connected through links (called link weights) that work used to solve a particular problem [96]. An ANN is designed to solve a specific problem, for instance, classification or regression problem, pattern recognition, through a process of learning from the training data. Generally, it is the process of adjusting the weights between neurons so that the error function value is minimal. The basic structure of an ANN usually consists of neurons grouped into input data layers, output data, and one or many hidden layers [97,98]. Based on the linking method, ANN can be classified into two main types: the recurrent neural network (RNN) and feedforward neural network (FNN). In particular, FNN is one of the most basic forms of artificial neural networks and is used successfully in many applications [99,100]. In an FNN, data are processed in a single direction, meaning that data from the input layer will only be transferred via hidden layers for calculation, and calculation results will be forwarded through the output layer to generate output data. The process of adjusting weights so that the network knows the relationship between the input and the desired output is called learning or training [101]. Currently, the mathematical algorithm used to adjust the performance of the FNN is now widely used as the backpropagation algorithm. The backpropagation algorithm uses a set of input and output values to find the desired neural network. A set of inputs is put into a certain preset system to calculate the output value, then this output value is compared with the actual value measured. If there is no difference, there is no need to perform a test. On the contrary, the weights will be changed during the backpropagation process to reduce the difference. The backpropagation network usually has one or more hidden layers with sigmoid-like neurons, and the output layer is neurons with linear transfer function [51]. However, in traditional BPNN networks, there are some shortcomings, such as slow convergence speed and easy falling to a local minimum [102]. In order to speed up the convergence rate and achieve higher accuracy, other training algorithms have been proposed and classified into three groups, namely the steepest descent, Quasi-Newton, and conjugate gradient. In this work, Matlab programming language (version 2018a [103]) has been employed for implementation of FNN.

#### 2.2.2. One Step Secant Method (OSS)

##### Quasi-Newton Method

The Newton method is based on the second-order Taylor series expansion. It is considered as an alternative algorithm to the conjugate gradient method, often used for fast optimization. For a given function *f*(*x*), Taylor’s series of *f*(*x*) around *x_k_* can be written as below [104,105]:

(1) $$f(x_{k}+\Delta x)\approx f(x_{k})+\nabla f{\left(x_{k}\right)}^{T}\Delta x+\frac{1}{2}\Delta x^{T}A\Delta x,$$

where *A* is an approximation of the Hessian matrix. The gradient of this approximation is:

(2) $$\nabla f(x_{k}+\Delta x)\approx \nabla f(x_{k})+A\Delta x,$$

We set this gradient to zero, thus:

(3) $$\Delta x=-A^{-1}\nabla f(x_{k})$$

In machine learning applications, the latter reflects the actual values of the weights, biases associated with the neurons [104]. The Newton algorithm is observed to achieve a faster convergence rate than that of the conjugate gradient methods. However, it is complex, and the computation cost of the Hessian matrix per iteration is expensive, especially in case of FNN. Later, a new class of algorithm based on the Newton method is proposed. This is called the quasi-Newton (secant) method, in which the computation of the second derivatives per step is avoided. The method lies in the update process of an approximation of the Hessian matrix by performing the computation as a function of the gradient. This algorithm requires more computation and storage per iteration than the conjugate gradient methods but generally converges in fewer iterations.

##### One Step Secant Algorithm

Given that the quasi-Newton algorithm demands more significant storage space and computation efforts, there is a need for a secant approximation that could avoid these disadvantages. The one-step secant (OSS) method is an effort to take advantage of the conjugate gradient and the quasi-Newton (secant) algorithms. In the OSS algorithm, the complete Hessian matrix does not need to be stored via an assumption that the previous Hessian is the identity matrix. It also provides an additional advantage that the actual search direction can be computed without inverting a matrix. In general, the OSS algorithm demands less storage and computation effort compared with the quasi-Newton algorithm per iteration, but slightly more than the conjugate gradient algorithm. The OSS algorithm could be considered as a compromise between the conjugate gradient algorithm and the full quasi-Newton algorithm.

#### 2.2.3. Prediction Performance Assessment

In this work, common statistical measurements, such as the coefficient of determination (*R*^2^), Mean Absolute Error (*MAE*), and Root Mean Square Error (*RMSE*) were used to assess and validate the FNN-OSS model. The *R*^2^ [106] allows identifying the statistical relationship between actual and output data. This measurement yields a value between 0 and 1 inclusive, in which 0 is referred to the case of no correlation, and 1 is referred to a total correlation. The formulation of *R*^2^ is [107,108]:

(4) $$R^{2}=\frac{\sum_{k=1}^{N}\left(p_{k}-\bar{p}\right)\left(w_{k}-\bar{w}\right)}{\sqrt{{\sum_{k=1}^{N}\left(p_{k}-\bar{p}\right)}^{2}{\sum_{k=1}^{N}\left(w_{k}-\bar{w}\right)}^{2}}},$$

where *N* is the number samples, $p_{k}$ and $\bar{p}$ are FNN-OSS output and mean FNN-OSS values, while $w_{k}$ and $\bar{w}$ are experimental and mean experimental values, respectively (k=1:N). In the case of Mean Absolute Error, the low value of *MAE* indicates good accuracy of prediction output using the models. *MAE* could be calculated using the following equation [109,110,111,112,113]:

(5) $$MAE=\frac{\sum_{k=1}^{N}\left|p_{k}-w_{k}\right|}{N},$$

where, $p_{k}$ and $w_{k}$ are predicted and observed values, respectively (k=1:N). The formulation of *RMSE* is described by the following equation [114]:

(6) $$RMSE=\sqrt{\frac{1}{N}\sum_{k=1}^{N}{\left(p_{k}-w_{k}\right)}^{2}}$$

Finally, the *Slope* criterion is defined as the *Slope* of the linear regression fit between predicted and observed vectors.

## 3. Results

### 3.1. Optimization of FNN-OSS Model

In this section, the optimization of the weight and bias parameters of FNN using OSS technique is presented. Table 4 indicates the characteristics of FNN. As shown in various studies in the literature, FNN with one hidden layer can solve many complex problems [115,116]. Therefore, in this study, FNN model with one hidden layer was finally chosen, and 20 neurons were found as the best number. With an architecture of 6-20-1, the model exhibited 120 weight parameters and 20 bias parameters in the hidden layer, 20 weight parameters and 1 bias parameter in the output layer. Hence, there were 161 parameters to be optimized, as indicated in Table 4. It is worth noticing that in this work, global optimization was adopted. The sigmoid function was chosen as an activation function for the hidden layer, whereas the linear function was selected as an activation function for the output layer [117]. The standard mean square error cost function was selected for the optimization problem. Finally, Table 5 indicates the description of the parameters of OSS used in this study.

**Figure 6:**
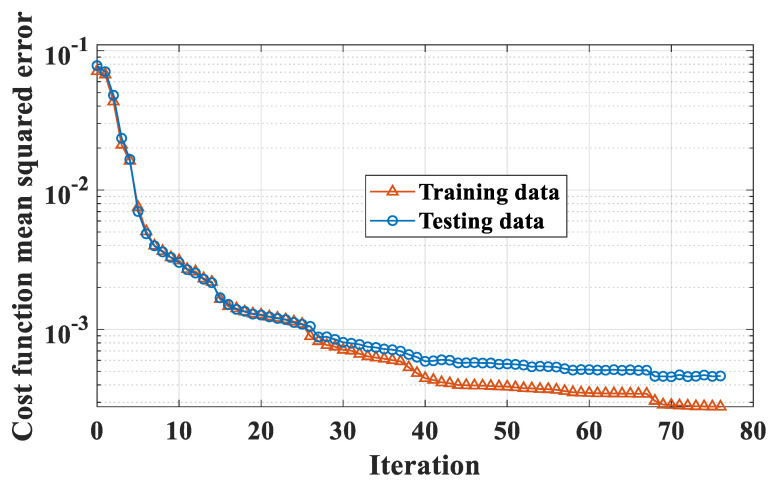
Evaluation of the cost function during optimization. The optimal iteration was 70.

The evaluation of cost function during the optimization process is presented in Figure 6, for both training and testing datasets. It should be noticed that the testing dataset was entirely new when applying the model. It is seen that a good evolution of mean square error for the testing dataset was obtained. In other words, there were no sudden changes during the optimization process. Finally, the optimal iteration was observed at 70, where the mean square error for the testing dataset started to increase [118]. The final configuration was used for performance analyses in the next sections.

### 3.2. Prediction Capability Assessment

#### 3.2.1. Global Analysis

**Figure 7:**
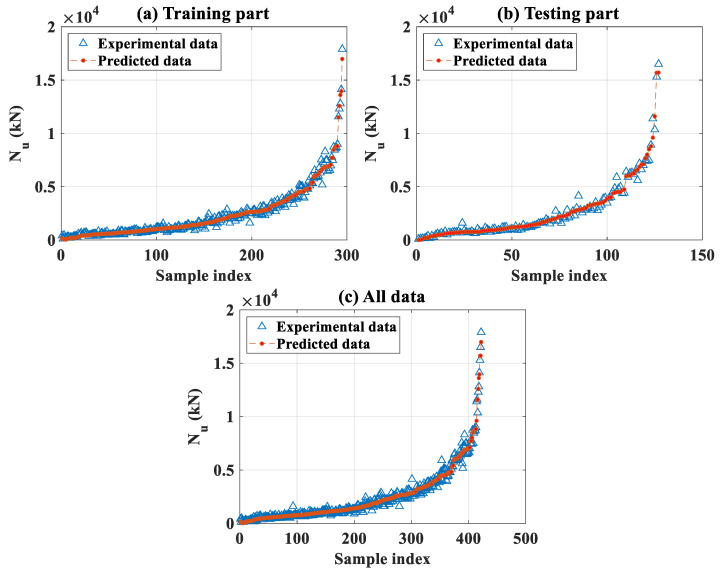
Comparison between experimental and output predicted data in a sorted mode for (**a**) training data, (**b**) testing data, and (**c**) all data.

The optimal FNN-OSS model identified in the previous section allowed predicting the axial capacity of the CFST columns for the training, testing, and all datasets. Figure 7a–c present the evolution of actual and predicted load-bearing capacity (*N_u_*) in a sorted mode for the training, testing, and all datasets, respectively. It is seen that the actual data located uniformly around the predicted one, i.e., no sign of over-or under-estimations, was observed.

**Figure 8:**
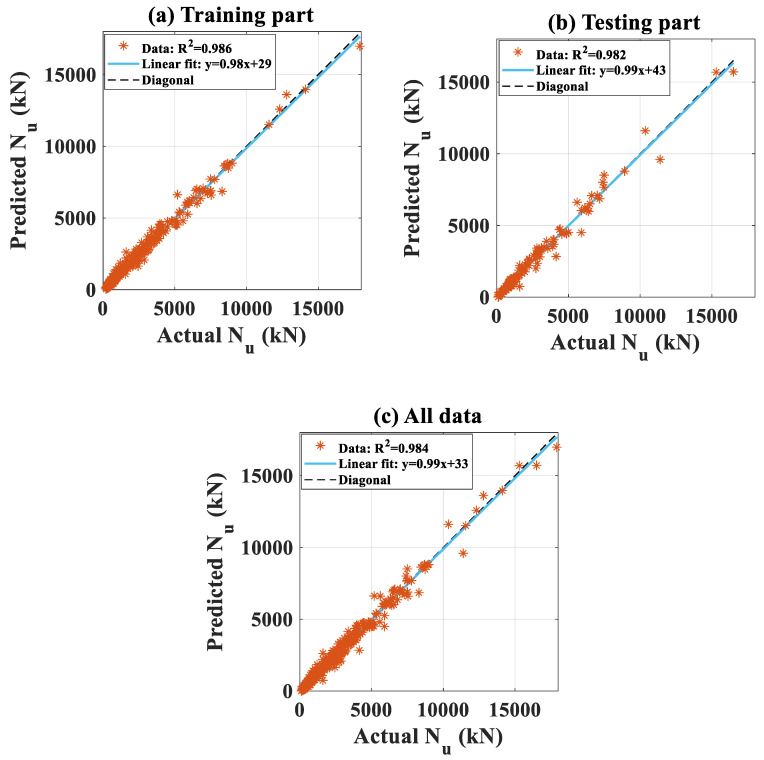
Comparison between experimental and output predicted data in regression scatter mode for (**a**) training data, (**b**) testing data, and (**c**) all data.

On the other hand, Figure 8a–c present the regression graphs of actual and predicted *N_u_* for the training, testing, and all datasets, respectively. Again, the data located uniformly around the diagonal line, showing that overfitting was prevented during the optimization process by using the OSS technique. Moreover, as observed in Figure 8 of regression, the values of the predicted axial capacity were not systematically too high or too low in the observation space.

As indicated in Table 6 for a summary of performance analyses, the values of *MAE* showed that the average magnitude of the residuals between the predicted and target data were 212.916, 245.159, and 222.620 kN, for the training, testing, and all datasets, respectively. The standard deviation of such residuals was demonstrated through *RMSE* values, which were 301.111, 380.354, and 326.985 kN, for the training, testing, and all datasets, respectively.

However, it is observed in both Figure 7 and Figure 8 that there were several extreme values of *N_u_* (i.e., higher than 8000 kN), which represented in a small number of data. These extreme values of *N_u_* could be considered as outliers and produced a higher value of *RMSE* than *MAE* (as the value of *RMSE* is sensitive to outliers). In terms of the coefficient of determination, the *R*^2^ were 0.986, 0.982, and 0.984 for the training, testing, and all datasets, respectively. These satisfying values confirmed the strong performance of the proposed FNN-OSS model. Finally, other error measurements such as *ErrorMean*, *ErrorStD*, and *Slope* are also indicated in Table 6, showing that a good agreement between the predicted and the actual values of axial capacity was obtained.

#### 3.2.2. Local Analysis

**Figure 9:**
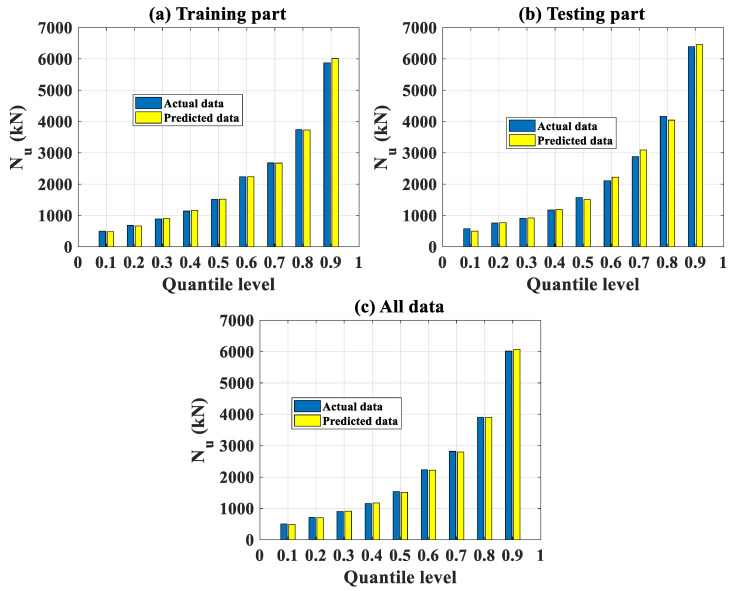
Comparison between experimental and output predicted data at different quantile levels of the distributions for (**a**) training data, (**b**) testing data, and (**c**) all data.

In this section, a local analysis of the prediction performance of the FNN-OSS model is presented. To this aim, nine quantile levels (from 10 to 90% with a step of 10%) of the probability density function of actual, predicted values of *N_u_* were identified. The results are plotted in Figure 9a–c for the training, testing, and all datasets, respectively. It is seen that the range of selected quantiles covered *N_u_* from about 500 to 6000 kN (corresponding to 10 and 90%, respectively). This point also confirmed that the number of extreme values of *N_u_* (i.e., higher than 8000 kN) was rather small, and the analysis herein allowed concentrating on the most representative data. It is seen that, locally, a good agreement between actual and predicted *N_u_* was obtained. Thus, it could be stated that the FNN-OSS model was efficient as proved at different quantile levels. Finally, the corresponding values of *N_u_* at each quantile levels are indicated in Table 7.

## 4. Discussion

### 4.1. Comparison of Performance

In this section, the performance of the deveveloped ANN-OSS model is compared with: (i) existing empirical equations in the literature and (ii) other machine learning models, when predicting the load-carrying capacity of rectangular CFST columns. In terms of existing emprirical equations, Han et al. [119] put forward the following equation for estimating the load-carrying capacity based on statistical analysis:

(7) $$N_{u}^{Han}=\left(1.18+0.85\frac{f_{y}A_{s}}{f_{c}^{\prime }A_{c}}\right)f_{c}^{\prime }A_{sc},$$

where *A_c_*, *A_s_*, *A_sc_* are the areas of the concrete core, the steel tube, and the total cross section, respectively. Similarly, Wang et al. [120] proposed the following equation:

(8) $$N_{u}^{Wang}=n_{a}f_{y}A_{s}+n_{c}f_{c}^{\prime }A_{c},$$

where *n_a_* and *n_c_* are as a function of material strength. In other study, Ding et al. [60] derived the following formulation for predicting the load-carrying capacity of CFST members:

(9) $$N_{u}^{Ding}=1.2f_{y}A_{s}+f_{c}^{\prime }A_{c},$$

On the other hand, several widely used regression machine learning models such as support-vector-machine (SVM) [121], fuzzy-logic (FL) [122] and ensemble boosted tree (EBT) [123] were trained to compare the prediction performance with the FNN-OSS model.

For an illustration purpose, a set of input data gathered from Refs. [65,79,85,86,90,92] was used and the values of inputs are indicated in Table 8 below, together with the experimental value of *N_u_*, as well as the prediction by using: (i) existing empirical equations (see Equations (7)–(9)); (ii) SVM, FL, EBT models; and (iii) FNN-OSS model. For a comparison purpose, an indicator Δ was computed as below:

(10) $$\Delta =\frac{N_{u}^{predicted}}{N_{u}^{\exp.}}\times 100,$$

where $N_{u}^{predicted}$ and $N_{u}^{\exp.}$ are the predicted and experimental values of *N_u_*, respectively. A summary of statistical analysis of Δ such as min, mean, max, standard deviation, and coefficient of variation is also provided at the end of Table 8 (the value of Δ is not shown in Table 8).

For the first six configurations in Table 8, it is seen that as empirical equations do not account the effect of the column’s length, thus the prediction by using Equations (7)–(9) exhibits the same values. Such a limitation is improved by using machine learning models, especially by using the FNN-OSS approach. The mean value of Δ is 128.3, 103.3, 120.4, 106.7, 107.3, 96.0 and 100.4% when using Han, Wang, Ding, SVM, FL, EBT and FNN-OSS models, respectively. Moreover, the FNN-OSS approach provides the best result in terms of coefficient of variation (10.2% compared to 31.1, 32.4, 30.7, 24.0, 26.4 and 11.2% of Han, Wang, Ding, SVM, FL, and EBT, respectively). From overall statistical performances, it could be concluded that the FNN-OSS model exhibits highest efficiency and performance in order to predict the load-carrying capacity of rectangular CFST columns. The performance comparison presented herein demonstrates that the machine learning technique can assist in the initial phase of the design of rectangular CFST members. In addition to a reliable prediction of load-carrying capacity, as presented above, FNN-OSS can also assist in the creation of load-carrying capacity continuous maps, within the ranges of the input variables adopted in this study.

### 4.2. Local Performance and Limitations

**Figure 10:**
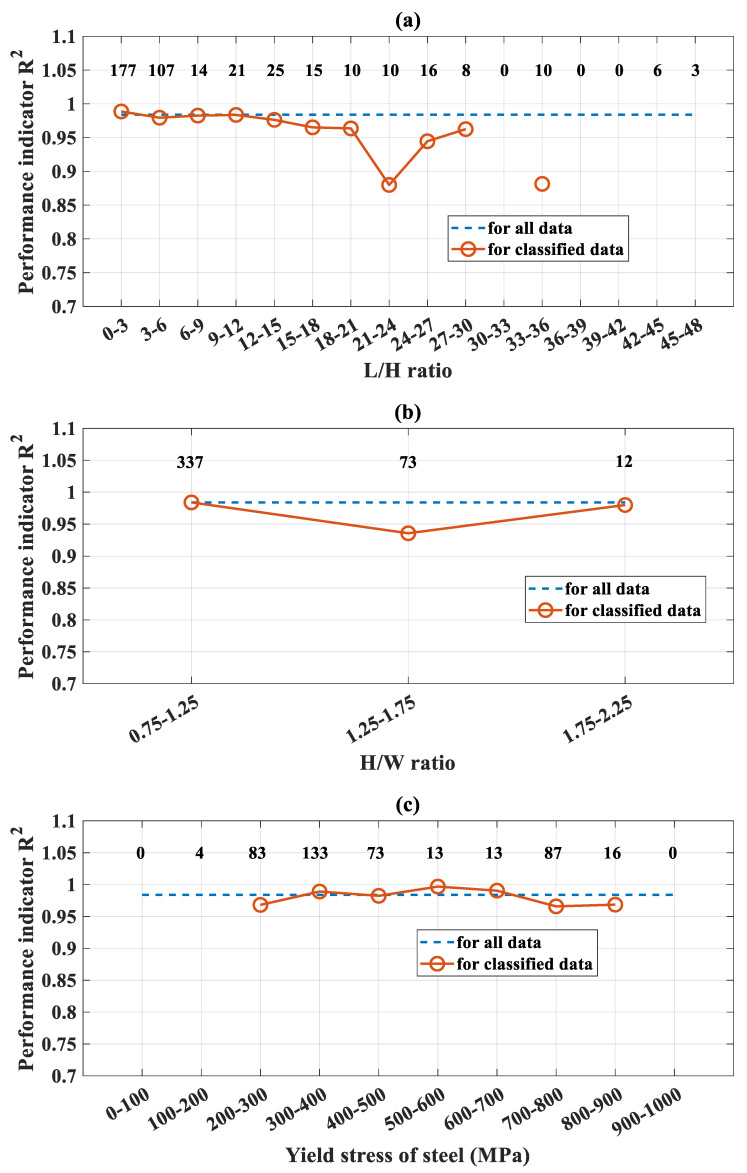

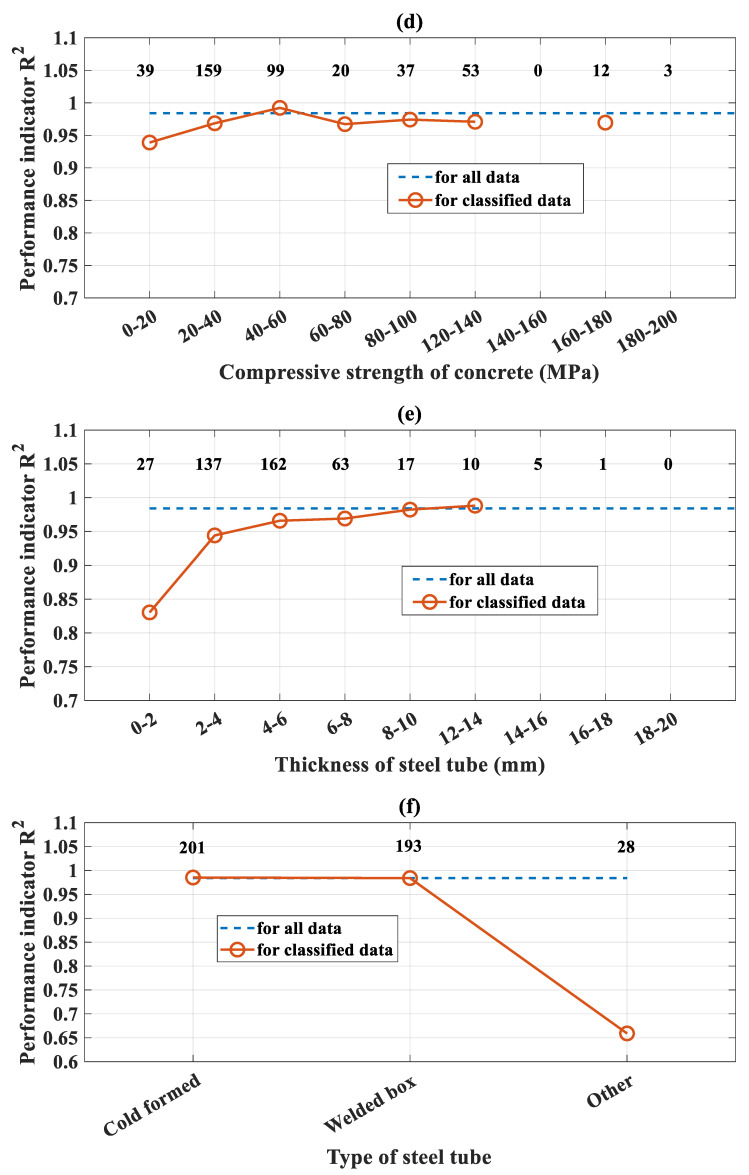
Details of the performance of FNN-OSS model at different classes of (**a**) *L/H* ratio, (**b**) *H/W* ratio, (**c**) yield stress of steel, (**d**) compressive strength of steel concrete, (**e**) thickness of steel tube, and (**f**) manufacturing type of steel tube. The number of data in each class is also indicated.

In this section, the performance and limitations of the FNN-OSS model are discussed from a structural engineering point of view. It should be noticed that there were 422 compressive test results collected in this study from the available literature. However, such a number of data might not guarantee that all the possible ranges could be covered. To reveal this problematic, the performance of the FNN-OSS model based on the coefficient of determination *R*^2^ was highlighted at different classes of variables. More specifically, Figure 10a–g show the *R*^2^ values in function of the length-to-height ratio, height-to-width ratio, yield strength of steel, compressive strength of concrete, thickness of steel tube, length of column, and manufacturing type of steel tube, respectively. In these figures, the number of data in each class was highlighted for better illustration. Histograms of variables could also be consulted in Figure 4 from the previous section.

As shown in Figure 10a, most of the data were classified as short and medium columns (i.e., 284/422 data, *L*/*H* ratio lower than 6). There were a few data for slender columns and no data for several values of *L*/*H* ratio. The values of *R*^2^ showed that the FNN-OSS model exhibited a good prediction capability for CFST columns with *L*/*H* ratio lower than 20, especially for the cases of *L*/*H* ratio lower than 10 (i.e., for short and medium columns). Nonetheless, considering the slender columns, a minimum performance of *R*^2^ of 0.87 was observed, compared to an *R*^2^ of 0.98 using all data.

Figure 10b highlights the performance of the FNN-OSS model in function of the shape of the cross-section. It is seen that for almost of data, the cross-section was square in shape (i.e., 337/422 data). However, the performance of the FNN-OSS model is quite strong for all classes, as the minimum value of *R*^2^ was about 0.94, as illustrated in Figure 10b. Nonetheless, more data should be collected for rectangular cross-sections as a perspective of this work to enhance the prediction performance of the model.

Figure 10c,d present the performance of the FNN-OSS model in function of mechanical strength of constituent materials, i.e., steel and concrete, respectively. It can be seen that the steel yield strength was mostly found in the range between 200 and 800 MPa, whereas the concrete compressive strength was varied between 20 and 140 MPa. In terms of prediction performance, the FNN-OSS model showed an excellent prediction capability for all classes of mechanical properties of the constituent materials. On the contrary, as pointed out in Figure 10d, not much data were collected for high strength concrete (i.e., higher than 70 MPa). Consequently, it is considered as a current limitation of the constructed model. It should be noticed that the concrete core exhibits a critical role in the composite CFST members, as it prevents the inward buckling of the steel tube [124,125].

Figure 10e presents the performance of the FNN-OSS model in function of the thickness of the steel tube. It is seen that there was not much data related to extremely thin-walled members (i.e., thickness inferior to 2 mm). Consequently, the performance of the FNN-OSS model for thin-walled structures was poor. However, for thickness superior to 2 mm, the performance of the prediction model was excellent, exhibiting a coefficient of determination higher than 0.95. As demonstrated in various studies in the literature, the thickness of the steel tube exposes a crucial role in the macroscopic behavior of the composite CFST columns [126,127]. Thus, this variable should be carefully investigated in further researches.

Finally, Figure 10f presents the performance of the FNN-OSS model in function of manufacturing types of steel tube. It is seen that almost rectangular steel tubes were manufactured as cold-formed or welded box (94% of the total data). As the number of classes is small (i.e., three classes), the performance of the FNN-OSS model was guaranteed. It could be concluded that the prediction model could work well for cold-formed and welded box tubes. However, for other types (28/422 data), the prediction performance was quite poor. This observation suggested that: (i) more data should be collected, and (ii) the manufacturing types of steel tube should be an input variable (i.e., categorical) in further studies.

## 5. Conclusions and Outlook

In this study, a consistent and effective machine learning algorithm was developed to estimate the load-bearing capacity of rectangular concrete-filled steel tubes. In particular, a novel hybrid machine learning model, based on a combination of feedforward neural network (FNN) and one step secant method (OSS), was proposed. Regarding the development and validation of the model, an experimental database containing 422 instances was gathered from the available literature, including six inputs representing the geometrical and material properties of rectangular concrete-filled steel tubes. Common statistical measurements, namely the coefficient of determination, mean absolute error, and root mean square error, were used for the assessment of the proposed machine learning model.

The model parameters of both FNN and OSS were first carefully selected, following by the local and global analysis on the prediction capability of the model. The results confirmed the effectiveness of the proposed FNN-OSS algorithm with excellent regression capability, i.e., *R*^2^ = 0.986, 0.982, and 0.984 for the training, testing, and all datasets. The advantages and limitations of the FNN-OSS model were finally given under a structural engineering point of view by analyzing the prediction performance with respect to different classes of input variables.

Overall, a robust machine learning algorithm for predicting the CFST load-bearing capacity was developed and thoroughly analyzed in this study. The results of this study might be useful for engineers and/or researchers to quick estimate the axial capacity of rectangular CFST columns, within the ranges of the input variables adopted in this study (see Table 2), and without the burden of the costly resources associated to finite element analysis. Moreover, the methodology proposed in this study can be applied to study other mechanical properties of CFST members based on experimental database. For instance the load-carrying capacity in the presence of loading eccentricity can be predicted if such an information can be gathered from the experimental tests. Similarly, the proposed appoach can be applied to predict the load-carrying capacity of CFST members with steel reinforcement, different cross-sectional shapes, or under fire loading, etc., if experimental database can be collected from the available literature. Finally, the prediction function can assist to the initial phase of design and analysis, before carrying out any laboratory experiments.

## Figures and Tables

**Table 1:** Database information and the corresponding number of data.

Nr.	Reference	Nr. of Data	% of Proportion	Nr.	Reference	Nr. of Data	% of Proportion
1	Aslani et al. [62]	12	2.8	20	Lin [63]	12	2.8
2	Bergmann [64]	4	0.9	21	Matsui & Tsuda [65]	6	1.4
3	Bridge [66]	1	0.2	22	Mursi & Uy [67]	4	0.9
4	Chapman & Neogi [68]	2	0.5	23	Sakino et al. [56]	48	11.4
5	Chen et al. [69]	4	0.9	24	Sakino et al. [70]	36	8.5
6	Ding et al. [60]	5	1.2	25	Schneider [26]	11	2.6
7	Du et al. [55]	14	3.3	26	Shakir-Khalil & Mouli [71]	14	3.3
8	Dundu [72]	27	6.4	27	Shakir-Khalil & Zeghiche [73]	1	0.2
9	Fong et al. [74]	1	0.2	28	Tao et al. [75]	2	0.5
10	Furlong [76]	5	1.2	29	Tomii & Sakino [77]	8	1.9
11	Ghannam et al. [78]	24	5.7	30	Uy [79]	18	4.3
12	Grauers [80]	18	4.3	31	Varma [81]	4	0.9
13	Han [82]	66	15.6	32	Vrcelj & Uy [83]	8	1.9
14	Han & Yang [84]	4	0.9	33	Xiong et al. [85]	15	3.6
15	Han & Yao [58]	34	8.1	34	Yamamoto et al. [86]	8	1.9
16	Han et al. [87]	2	0.5	35	Yang & Han [88]	2	0.5
17	Khan et al. [89]	55	13.0	36	Yu et al. [90]	10	2.4
18	Knowles & Park [91]	6	1.4	37	Zhu et al. [92]	6	1.4
19	Lam & Williams [93]	15	3.6		Total	422	100.0

**Table 2:** Initial statistical analysis of the database.

Parameter	Height of Steel Tube (*H*)	Width of Steel Tube (*W*)	Thickness of Steel Tube (*t*)	Length of Column (*L*)	Yield Stress of Steel (*f_y_*)	Compressive Strength of Concrete (*f’_c_*)	Load-Bearing Capacity (*N_u_*)
Unit	mm	mm	mm	mm	MPa	MPa	kN
Minimum	60.00	60.00	0.70	60.00	194.00	7.90	105.40
Q25	101.60	100.00	3.00	450.00	320.00	26.39	808.08
Median	149.35	120.00	4.80	634.00	384.65	40.91	1537.00
Q75	195.00	160.00	5.48	1514.00	618.00	80.00	3183.00
Maximum	500.00	500.00	16.00	4500.00	835.00	164.10	17,900.00
Mean	154.02	141.72	4.80	1115.58	459.11	54.36	2500.92
StD	66.08	65.36	2.34	957.49	195.18	37.00	2602.94
CV (%)	42.90	46.12	48.87	85.83	42.51	68.06	104.08

**Table 3:** Details of classification of *L*/*H* ratio, *H*/*W* ratio, and type of steel tube.

N°	*L*/*H* Ratio	Number	*H*/*W* Ratio	Number	Steel Tube Type	Number
1	0–3	177	0.75–1.25	337	Cold formed	201
2	3–6	107	1.25–1.75	73	Welded box	193
3	6–9	14	1.75–2.25	12	Other	28
4	9–12	21	Total	422	Total	422
5	12–15	25				
6	15–18	15				
7	18–21	10				
8	21–24	10				
9	24–27	16				
10	27–30	8				
11	30–33	0				
12	33–36	10				
13	36–39	0				
14	39–42	0				
15	42–45	6				
16	45–48	3				
	Total	422				

**Table 4:** FNN’s characteristics.

Parameter	Notation	Value and Description
Neurons in input layer	n_input_	6
Number of hidden layer	n_layer_	1
Neurons in hidden layer	n_neuron_	20
Neurons in output layer	n_output_	1
Number of weight parameters in hidden layer		n_input_ × n_neuron_ = 120
Number of bias parameters in hidden layer		n_neuron_ (20)
Number of weight parameters in output layer		n_neuron_ × n_output_ = 20
Number of bias parameters in output layer		n_output_ (1)
Number of total parameters to be optimized		161
Training algorithm	OSS	One Step Secant
Cost function	MSE	Mean square error
Activation function for hidden layer		Sigmoid
Activation function for output layer		Linear

**Table 5:** Parameters of OSS used in this study.

Parameter	Value and Description
Initial step size	0.01
Maximum step size	26
Search routine	linear
Lower limit	0.1
Upper limit	0.5
Scale tolerance	20
Maximum validation checks	6
Minimum gradient	10^−10^
Maximum iteration	1000

**Table 6:** Performance indicators of the optimal FNN-OSS model.

Indicator	Training Part	Testing Part	All Data
*R* ^2^	0.986	0.982	0.984
*RMSE*	301.111	380.354	326.985
*MAE*	212.916	245.159	222.620
*ErrorMean*	8.605	−10.279	2.922
*ErrorStD*	301.499	381.721	327.360
*Slope*	0.984	0.988	0.986
*SlopeAngle*	44.552	44.650	44.591

**Table 7:** Comparison of different quantile levels between predicted and actual data.

Quantile	Training Actual	Predicted	Ratio	Testing Actual	Predicted	Ratio	All Data Actual	Predicted	Ratio
0.1	501.71	478.92	0.955	577.80	502.51	0.870	505.59	494.66	0.978
0.2	682.00	666.38	0.977	759.93	766.54	1.009	716.37	702.09	0.980
0.3	890.00	903.50	1.015	909.00	920.13	1.012	899.69	912.10	1.014
0.4	1142.00	1164.17	1.019	1174.91	1193.35	1.016	1155.64	1175.72	1.017
0.5	1516.26	1520.30	1.003	1572.00	1510.30	0.961	1537.00	1515.30	0.986
0.6	2237.00	2234.19	0.999	2108.70	2222.24	1.054	2232.80	2222.24	0.995
0.7	2680.00	2673.99	0.998	2882.00	3093.02	1.073	2817.90	2795.47	0.992
0.8	3743.50	3732.15	0.997	4164.90	4045.63	0.971	3902.00	3899.69	0.999
0.9	5873.00	6018.26	1.025	6393.00	6453.91	1.010	6014.50	6074.74	1.010
		Average	0.999		Average	0.997		Average	0.997

**Table 8:** Comparison of performance between FNN-OSS, existing equations in the literature and other prediction models.

*H* (mm)	*W* (mm)	*t* (mm)	*L* (mm)	*f_y_* (Mpa)	*f’_c_* (Mpa)	Exp. *N_u_* (kN)	Predicted by Proposed Equation of Han et al. 2005 [119] (kN)	Predicted by Proposed Equation of Wang et al. 2017 [120] (kN)	Predicted by Proposed Equation of Ding et al. 2014 [60] (kN)	Predicted by SVM (kN)	Predicted by FL (kN)	Predicted by EBT (kN)	Predicted by FNN-OSS (kN)	Ref.
150	150	4.5	600	438.4	31.9	1623.1	1950.7	1674.0	2011.4	2134.2	2242.4	1282.6	1690.7	[65]
150	150	4.5	1200	438.4	31.9	1611.2	1950.7	1674.0	2011.4	2013.9	2107.3	1266.9	1613.8	
150	150	4.5	1800	438.4	31.9	1598.2	1950.7	1674.0	2011.4	1893.5	1972.1	1266.9	1549.2	
150	150	4.5	2700	438.4	31.9	1378.0	1950.7	1674.0	2011.4	1712.9	1769.4	1239.5	1446.1	
150	150	4.5	3600	438.4	31.9	1161.8	1950.7	1674.0	2011.4	1532.3	1566.7	1152.4	1253.5	
150	150	4.5	4500	438.4	31.9	923.7	1950.7	1674.0	2011.4	1351.7	1363.9	1152.4	1091.0	
100.2	100.2	2.18	300.6	300.0	25.7	609.0	542.7	461.6	543.8	165.6	−108.9	621.1	492.3	[86]
200.3	200.3	4.35	600.9	322.0	29.6	2230.0	2421.2	2050.0	2404.1	2907.4	3314.9	2247.6	2262.0	
300.5	300.5	6.1	901.5	395.0	26.5	5102.0	5443.9	4682.0	5607.5	5535.8	6561.6	4467.1	4835.7	
100.1	100.1	2.18	300.3	300.0	53.7	851.0	872.9	712.4	799.6	590.7	376.3	791.2	708.9	
200.1	200.1	4.35	600.3	322.0	57.9	3201.0	3754.5	3062.8	3437.2	3334.9	3802.7	2790.6	3231.9	
300.7	300.7	6.1	902.1	395.0	52.2	6496.0	8191.4	6782.8	7751.9	5932.0	7014.5	6038.7	6509.3	
100.1	100.1	2.18	300.3	300.0	61.0	911.0	959.3	778.0	866.5	702.0	503.4	816.6	775.9	
200.3	200.3	4.35	600.9	322.0	63.7	3417.0	4035.5	3276.8	3655.9	3427.5	3909.0	2828.7	3435.8	
110	110	5	330	784.2	28.0	1836.0	2093.5	1837.1	2256.2	2081.5	1874.7	2101.4	1913.9	[79]
110	110	5	330	784.2	28.0	1832.0	2093.5	1837.1	2256.2	2081.5	1874.7	2101.4	1913.9	
160	160	5	480	784.2	30.0	2868.0	3257.3	2918.5	3592.2	3150.2	3212.3	3046.3	2824.6	
160	160	5	480	784.2	30.0	2922.0	3257.3	2918.5	3592.2	3150.2	3212.3	3046.3	2824.6	
210	210	5	630	784.2	32.0	3710.0	4678.3	4149.1	5138.3	4219.0	4549.9	3613.9	3894.0	
210	210	5	630	784.2	32.0	3483.0	4678.3	4149.1	5138.3	4219.0	4549.9	3613.9	3894.0	
100	100	1.9	300	404.0	96.1	1209.0	1410.2	1136.9	1250.5	1366.2	1183.2	1005.1	1044.1	[90]
100	100	1.9	300	404.0	96.1	1220.0	1410.2	1136.9	1250.5	1366.2	1183.2	1005.1	1044.1	
100	100	1.9	300	404.0	96.1	1190.0	1410.2	1136.9	1250.5	1366.2	1183.2	1005.1	1044.1	
100	100	1.9	300	404.0	96.1	1220.0	1410.2	1136.9	1250.5	1366.2	1183.2	1005.1	1044.1	
100	100	1.9	900	404.0	96.1	1013.0	1410.2	1136.9	1250.5	1245.8	1048.1	979.8	1140.7	
100	100	1.9	900	404.0	96.1	1010.0	1410.2	1136.9	1250.5	1245.8	1048.1	979.8	1140.7	
100	100	1.9	1500	404.0	96.1	915.0	1410.2	1136.9	1250.5	1125.4	912.9	979.8	1225.9	
100	100	1.9	1500	404.0	96.1	945.0	1410.2	1136.9	1250.5	1125.4	912.9	979.8	1225.9	
100	100	1.9	3000	404.0	96.1	474.0	1410.2	1136.9	1250.5	824.4	575.0	488.7	478.9	
100	100	1.9	3000	404.0	96.1	466.0	1410.2	1136.9	1250.5	824.4	575.0	488.7	478.9	
150	150	8	450	779.0	152.3	6536.0	7813.8	6055.8	6982.4	5572.8	6060.2	6403.0	7010.3	[85]
150	150	8	450	779.0	157.2	6715.0	7943.9	6142.0	7070.4	5647.6	6145.6	6403.0	6916.8	
150	150	8	450	779.0	147.0	6616.0	7673.1	5962.5	6887.3	5492.0	5967.9	6375.1	7079.1	
150	150	8	450	779.0	164.1	7276.0	8127.1	6263.5	7194.3	5752.8	6265.8	6403.0	6739.8	
150	150	8	450	779.0	148.0	6974.0	7699.6	5980.1	6905.2	5507.2	5985.3	6403.0	7068.8	
150	150	12	450	756.0	152.3	8585.0	10,076.1	7174.7	8427.2	6561.1	7338.7	7837.1	8722.7	
150	150	12	450	756.0	157.2	8452.0	10,206.2	7250.9	8505.0	6635.8	7424.1	7837.1	8633.5	
150	150	12	450	756.0	147.0	8687.0	9935.4	7092.2	8343.1	6480.2	7246.4	7723.4	8802.9	
150	150	12	450	756.0	164.1	8730.0	10,389.4	7358.3	8614.5	6741.1	7544.3	7837.1	8477.2	
150	150	12	450	756.0	148.0	8912.0	9962.0	7107.8	8358.9	6495.5	7263.8	7837.1	8789.0	
150	150	12.5	450	446.0	152.3	5953.0	7796.7	5212.4	6059.2	6082.8	7015.5	6100.8	6055.0	
150	150	12.5	450	446.0	157.2	5911.0	7926.8	5287.4	6135.8	6157.5	7100.9	6100.8	6042.5	
150	150	12.5	450	446.0	147.0	6039.0	7655.9	5131.2	5976.4	6002.0	6923.2	6015.0	6036.6	
150	150	12.5	450	446.0	164.1	6409.0	8109.9	5393.0	6243.6	6262.8	7221.1	6100.8	5980.2	
150	150	12.5	450	446.0	148.0	6285.0	7682.5	5146.5	5992.0	6017.2	6940.6	6100.8	6042.6	
197	197	6.4	600	437.9	21.0	2730.0	3038.9	2665.7	3276.4	3462.5	3933.0	3032.7	2879.8	[92]
198.5	198.5	6.1	600	437.9	20.4	3010.0	2931.7	2581.4	3174.5	3407.7	3863.9	2919.2	2808.2	
200.5	200.5	6.3	600	437.9	19.3	2830.0	2991.0	2639.0	3254.0	3486.0	3964.7	2919.2	2865.5	
201	201	10.3	600	381.7	21.0	3980.0	4165.4	3459.7	4281.9	4445.3	5233.3	3955.0	3689.9	
201	201	10	600	381.7	20.4	3920.0	4028.7	3366.3	4167.2	4358.5	5124.1	3955.0	3567.5	
199.5	199.5	10.1	600	381.7	19.3	3900.0	3981.2	3325.7	4126.1	4336.2	5098.5	3685.4	3542.5	
						Min of Δ	89.1	75.8	89.3	27.2	−17.9	78.6	80.8	
						Mean of Δ	128.3	103.3	120.4	106.7	107.3	96.0	100.4	
						Max of Δ	302.6	244.0	268.3	176.9	148.7	124.8	134.0	
						StD of Δ	40.0	33.5	37.0	25.6	28.4	9.8	10.2	
						CoV of Δ	31.1	32.4	30.7	24.0	26.4	10.2	10.2	
